# Comprehensive Molecular Evaluation of HNF-1 Alpha, miR-27a, and miR-146 Gene Variants and Their Link with Predisposition and Progression in Type 2 Diabetes Patients

**DOI:** 10.3390/jpm13081270

**Published:** 2023-08-17

**Authors:** Rashid Mir, Imadeldin Elfaki, M. E. Elangeeb, Mamdoh S. Moawadh, Faris Jamal Tayeb, Jameel Barnawi, Ibrahim Altedlawi Albalawi, Amnah A. Alharbi, Marwan H. Alhelali, Basim S. O. Alsaedi

**Affiliations:** 1 Department of Medical Lab Technology, Prince Fahad Bin Sultan Chair for Biomedical Research, Faculty of Applied Medical Sciences, University of Tabuk, Tabuk 71491, Saudi Arabia; mmoawadh@ut.edu.sa (M.S.M.); f.tayeb@ut.edu.sa (F.J.T.); jbarnawi@ut.edu.sa (J.B.); 2 Department of Biochemistry, Faculty of Science, University of Tabuk, Tabuk 47512, Saudi Arabia; ahalharbi@ut.edu.sa; 3 Department of Basic Medical Sciences, Faculty of Applied Medical Sciences, University of Bisha, Bisha 67714, Saudia Arabia; melnageeb@ub.edu.sa; 4 Department of Surgical Oncology, Faculty of Medicine, University of Tabuk, Tabuk 47713, Saudi Arabia; drbalawi@yahoo.com; 5 Department of Statistics, University of Tabuk, Tabuk 47512, Saudi Arabia; malhilaly@ut.edu.sa (M.H.A.); balsaedi@ut.edu.sa (B.S.O.A.)

**Keywords:** single-nucleotide variations (SNVs), Amplification Refractory Mutation System PCR (ARMS-PCR), microRNAs, miR-27a rs895819 SNV, miR-146 rs2910164, hepatocytes nuclear factor-1 alpha (HNF-1 alpha), type 2 diabetes

## Abstract

Background: Type 2 diabetes (T2D) is a metabolic condition induced by insulin resistance and pancreatic beta cell dysfunction. MicroRNAs (miRNAs) have biological significance because they regulate processes such as the molecular signaling pathways involved in the pathophysiology of diabetes mellitus. The hepatocyte nuclear factor-1 alpha (HNF-1 alpha) is a transcription factor found in hepatocytes and the pancreas. Mutations in the HNF-1 alpha gene were reportedly associated with maturity-onset diabetes of the young (MODY). The objective of the present study was to examine the associations between MiR-27a, MiR-146, and HNF-1 alpha single-nucleotide variations (SNVs) with T2D risk in the Saudi population. Methodology: We evaluated the association of SNVs of miR-27a rs895819 A>G, 146a-rs2910164 C>G, and HNF-1 alpha rs1169288 G>T (I27L) with the risk of T2D in Saudi patients with the Amplification Refractory Mutation System PCR (ARMS-PCR). For the miR-27a SNVs, we used 115 cases (82 males, 33 females) and 117 matched healthy controls (HCs); for the Mir-146 SNVs, we used 103 cases (70 males, 33 females) and 108 matched HCs; and for the HNF-1 alpha, we employed 110 patients (80 males, 30 females) and 110 HCs. The blood biochemistry of the participants was essayed using commercial kits, and the methods of statistical analysis used were the Chi-square test, the Fisher exact test, and a multivariate analysis based on logistic regression, like the odds ratio (OD) and risk ratio (RR), with 95% confidence intervals (CIs). Results: The MiR-27a rs895819 AG genotype was linked to increased T2D susceptibility, with OR = 2.01 and *p*-value = 0.011, and the miR-146 rs2910164 CG genotype and C allele were linked to an elevated risk of T2D, with OR = 2.75, *p*-value < 0.0016, OR = 1.77, and *p*-value = 0.004. The results also showed that the GT genotype and T allele of the HNF-1 alpha (rs1169288) G>T is linked to T2D, with OR = 2.18, *p*-value = 0.0061, and 1.77, *p*-value = 0.0059. Conclusions: The SNVs in miR-27a, miR-146, and HNF-1 alpha can be potential loci for T2D risk. The limitations of this study include the relatively small sample size and the fact that it was a cross-sectional study. To our knowledge, this is the first study to highlight the association between miR-27a, miR-146, and HNF-1 alpha SNVs and the risk of T2D in the Saudi population. Future large-scale case–control studies, as well as studies on the functions of the proteins and protein interaction studies for HNF-1 alpha, are required to verify our findings. Furthermore, these findings can be used for the identification and stratification of at-risk populations via genetic testing for T2D-prevention strategies.

## 1. Introduction

Both the incidence and prevalence of diabetes mellitus (DM) have increased all over the world in recent decades [1]. Diabetes mellitus is a metabolic disorder characterized by increased blood sugar. It can cause significant complications such as blindness (diabetic retinopathy), renal failure (diabetic nephropathy), nerve damage (diabetic neuropathy), cardiovascular disease (CVD), and limb amputation due to diabetic foot ulcers [1]. In addition to these complications, diabetes has a very serious socioeconomic impact [1,2,3]. Individuals with type 2 diabetes mellitus (T2D) account for more than 90% of the total number of DM patients [4]. The prevalence of T2D is increasing all over the world, including in the KSA. In the KSA, the prevalence has increased in the last 20 years [5]. The cause of T2D is insulin resistance or defective insulin action in peripheral tissues, hepatocytes, muscles, and fat tissues [6]. Insulin resistance is accompanied by compensatory insulin release from the pancreatic beta cells, which eventually leads to beta cell exhaustion and dysfunction [7]. The traditional risk factors for T2D include a sedentary lifestyle, obesity, poor diet, smoking, gestational diabetes mellitus, and genetic risk factors [8]. Genome-wide association studies (GWAs) have demonstrated the link between certain loci and diseases such as cancer, DM, CVD, and others [9,10,11,12,13,14,15,16,17,18].

**Figure 1:**
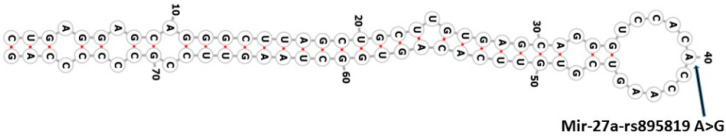
The structure of miR-27a showing the position of the MicroR-27-rs895819 A>G SNV. This figure was prepared using the webserver http://rna.tbi.univie.ac.at/cgi-bin/RNAWebSuite/RNAfold.cgi (accessed on: 2 October 2022).

**Figure 2:**
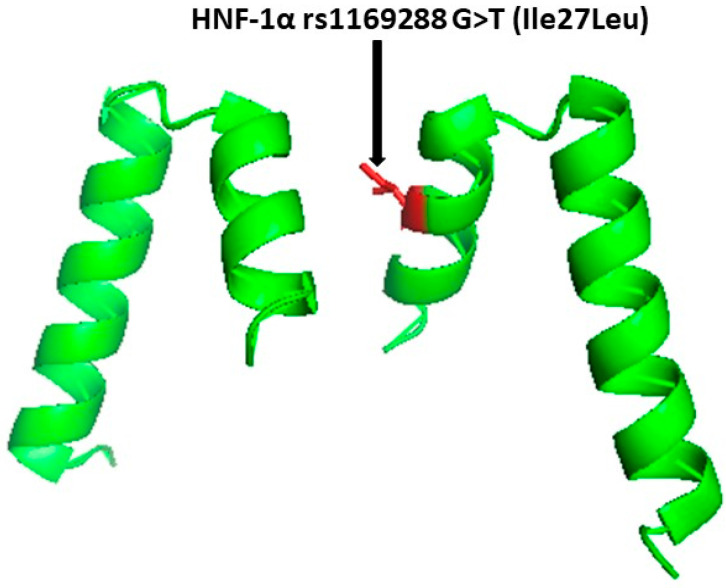
The cartoon protein structure (PDB: 2GYP) of the *HNF1A* showing the site of the HNF-1 alpha (rs1169288) A>C mutation that results in the transformation of the amino acid Isoleucine 27 to leucine. The site of amino acid substitution is shown in red. This figure was prepared using PyMOL.

MicroRNAs (miRNAs) are short non-coding ribonucleic-acid molecules that regulate the transcription of genes involved in crucial physiological processes [19,20]. The miR-27a is reportedly involved in insulin resistance and glucose metabolism via the PPAR-γ-mediated PI3K/Akt signaling pathway, and miR-27a is a potential target for decreasing insulin resistance and sugar metabolism in T2D [21]. The MiR-27a rs895819 A>G (Figure 1) is located on chromosome 19, and it has been associated with risk of cardiovascular disease and cancer among the Chinese population [22,23,24]. The miR-146a is involved glucose and lipid metabolism via the regulation of genes involved in insulin sensitivity and lipogenesis [25]. The gene polymorphism of the miR-146a rs2910164 C>G is associated with metabolic syndrome [26]. The hepatocyte nuclear factor-1 alpha (HNF-1 alpha) is a transcription factor expressed in hepatocytes, the gut, the pancreas, and renal cells [27]. It is involved in the growth and physiology of pancreatic beta cells and reportedly expressed in the growth stage of pancreatic cells [28]. The HNF-1 alpha was reportedly involved in the secretion of insulin and the metabolism of lipids and proteins, as well as in the renal reabsorption of glucose [27]. The gene variants in the *HNF1* alpha were associated with T2D and glycemic features in several populations, as well as in maturity-onset diabetes of the young (MODY) [28,29]. In the current study, we investigated the associations between miR-27-rs895819 A>G (Figure 1), miR-146 rs2910164 C>G, and HNF-1 alpha rs1169288 G>T (Figure 2) single-nucleotide variations (SNVs) and the induction of T2D in the Saudi population. Through genetic testing, the verification of the associations of these SNVs with the induction of T2D will help to identify individuals who are at risk of developing T2D. This could be very useful in delaying or preventing T2D, as these SNVs may represent additional T2D biomarkers [30,31].

Figure 1: The site of the SNV indicated in the structure of miR-27a.

Figure 2: The secondary structure of the *HNF1* alpha showing the site of the amino acid change.

## 2. Methodology

### 2.1. Subjects of the Study

The present research study received ethical approval from the University of Tabuk and Armed Forces hospitals (C2016-115). The T2D cases were extracted from the OPD of the different hospitals in the Tabuk city. The WHO criteria were used to diagnose T2D cases [32]. For the genotyping of the miR-27a rs895819 A>G SNV, we used 115 T2D cases and 117 HCs. For the Mir-146 rs2910164 C>G SNV, we used 103 T2D cases and 108 HCs.

#### 2.1.1. Inclusion Exclusion Criteria for T2D Cases

The T2D patients selected were ethnic citizens (males and females) of Saudi Arabia. All the T2D patients provided informed written consent prior to the collection of the samples. We excluded patients with any significant chronic disease (e.g., malignancy or hyperlipidemia).

#### 2.1.2. Inclusion Exclusion Criteria for Controls

The general population of the same geographic area served as the source of the controls. Blood chemistry and hematology tests were performed as part of routine clinical procedures. The controls that appeared to be healthy and had no serious illnesses were chosen as HCs.

### 2.2. Sample Collection and Extraction of Genomic DNA

Each patient and control individual had a venipuncture to draw a three to four milliliter sample of peripheral blood into EDTA tubes. Using the DNeasy Blood Kit (Qiagen, Germany), genomic DNA extraction was carried out in accordance with the given manufacturer instructions. Following purification, the DNA was stored at −20 °C until genotyping.

### 2.3. Genotyping of miR-27a, miR-146, and HNF1alpha (rs1169288) SNVs

For the genotyping of the SNVs under study, we employed the Amplification Refractory Mutation System PCR (ARMS-PCR). This is a simple, rapid, reliable, efficient, and inexpensive method that has been successfully used in many studies [33,34]. For miR-27a- rs895819 SNV genotyping, we used the primers reported in a study by Mashayekhi et al., 2018 [35]. For miR-146 rs2910164 SNV genotyping, we used the primers reported in a study by Mir et al., 2020 [36]. The primers for the *HNF1* alpha rs1169288 G>T, and all primers for genotyping of other SNVs are shown in Table 1.

### 2.4. Genotyping of the HNF1alpha (rs1169288) A>C (I27L), miR-27a rs895819 A>G, and miR-146a-rs2910164 C>G

ARMS-PCR reaction of a 12 μL sample containing DNA (60 ng), Fo—0.13 μL, Ro—0.13 L, FI—0.15 L, RI—0.15 μL (25 pmol of required primer), and 7 μL of Green Master Mix (2X) (Cat# M712C, Promega, Madison, WI, USA) was used for the PCR reaction. Then, to make the entire volume, 12 μL of nuclease-free H2O2 was added. Primer3 software was used to build ARMS primers for *HNF1A* (rs1169288) A>C (I27L), miR-27a (rs895819 A>G), and miR-146a (rs2910164 C>G), respectively.

#### 2.4.1. PCR Programming

Gradient PCR is a technique that can be used to achieve the ideal annealing temperature within a single experimental setup, omitting multiple procedures. The best results were obtained using a gradient PCR thermocycler at temperatures between 55 °C and 64 °C for *HNF1A* A>C (I27L) (62 °C), 63.5 °C for miR-27a rs895819 A>G, and 62.5 °C for miR-146a-rs2910164 C>G. The yields of all three PCR products increased as the number of cycles was reduced from 35 to 30. Initial denaturation was carried out at 95 °C for 10 min, followed by 32 cycles of denaturation at 95 °C for 35 s. The annealing temperatures for 35 s were 62 °C for *HNF1A* (rs1169288) A>C (I27L) (63 °C), 63 °C for miR-27a rs895819 A>G, and 62 °C for miR-146a-rs2910164 C>G.

#### 2.4.2. Visualization of the PCR Product and Gel Electrophoresis

The amplified PCR products were separated using agarose gel electrophoresis (2.5%), 0.5 g/mL EtBr, and a UV transilluminator to visualize the results.

#### 2.4.3. Amplification of microRNA-27a rs895819 A>G SNP

**Figure 3:**
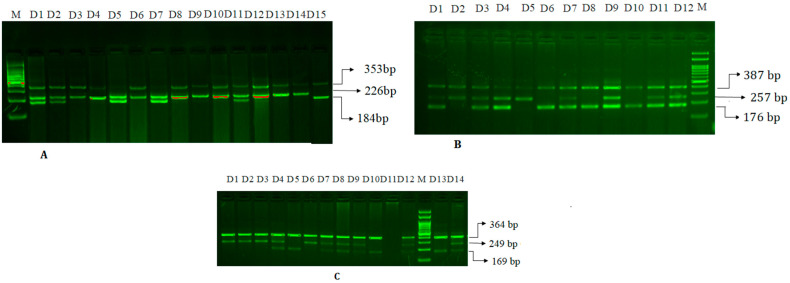
Agarose gel electrophoresis for the genotyping of the SNVs by ARMS-PCR. (**A**). MiR-27a rs895819 A>G: Legend-M-100 bp DNA ladder, Homozygous-GG (184 bp)-D15, Homozygous-AA (226 bp)-D3, D4, D6, D8, D9, D10, D12, D13, D14, Heterozygous-D1, D2, D5, D7, D11. (**B**) HNF-1 alpha rs1169288 G>T (Ile27Leu): Legend-M-100 bp DNA ladder: Homozygous-AA (257 bp)-D2, D5 Homozygous-GG (176 bp)-D6, D8, D10: Heterozygous-D1, D3, D4, D7, D9, D10, D11. (**C**): MiR-146 rs2910164 C>G: Legend-M-100 bp DNA ladder, Homozygous-GG (249 bp)-D1, D2, D3, D6 Homozygous-CC (169 bp)-D5, D10, D13, Heterozygous-D4, D7, D8, D9, D12, D14.

The miR-27′s outer region was amplified by the outer primers FO and RO, yielding a band of 353 bp, which proved useful in a DNA purity check. A band of 226 bp was produced by primers FI and R2 amplifying the A allele, and a band of 184 bp was produced by primers F2 and R1 amplifying the G allele, as depicted in Figure 3A.

Figure 3: Genotyping of the SNVs using ARMS-PCR.

#### 2.4.4. Amplification of *HNF1A* (rs1169288) A>C (I27L) SNP

The *HNF1A* rs1169288) A>C (I27L) gene’s primers—Fo and Ro—were amplified to create a band of 387 bp, which helped to check the quality and quantity of the DNA. The primers FI and Ro amplified a band of 176 base pairs (bp) for the C allele, and the primers Fo and RI amplified a band of 257 base pairs (bp) for the A allele, as depicted in Figure 3B.

#### 2.4.5. Amplification of miR-146a rs2910164 C>G SNP

As a qualitative and quantitative DNA experimental control, the primers Fo/Ro flank the miR-146a-rs2910164 C>G gene’s exon and amplify into a band of 364 bp. A band of 169 base pairs (bp) was produced by the amplification of the C allele (wild-type allele) by primers FI/Ro, while a band of 249 base pairs (bp) was produced by the amplification of the mutant G allele by primers Fo/RI, as depicted in Figure 3C.

### 2.5. Statistical Analyses

To compare the SNV genotype distribution between cases and controls and compare genotypes with other clinic-pathological characteristics, Chi-square analysis and Fisher’s exact test were used. The measured genotype frequencies within the control were compared using Student’s *t*-test (*p*-values = 0.05) to determine whether Hardy–Weinberg equilibrium was obeyed or not. We performed multivariate analyses to compare odds ratios (ORs), risk ratios (RRs), and risk differences (RDs) with 95% confidence intervals (CIs) in order to investigate the connection between SNVs and susceptibility to T2D. To determine the relationship between single-nucleotide variation genotypes and risk to T2D, a multivariate analysis based on logistic regression was performed. Odds ratios (ORs) and risk ratio (RRs) with 95% confidence intervals (CI) were calculated for each group.

## 3. Results

### 3.1. Demographic Characteristics of T2D Patients

There were 232 participants in this case–control study, 115 of whom had clinically proven T2D, and 117 of whom were healthy controls (Table 2). Table 2 provides a summary of all the demographic data for the 232 T2D patients and healthy control participants who underwent treatment in sequence. Full clinical data for 115 people were available. Based on their ages and genders, T2D patients were divided into two groups: those under 40 and those over 40 (82 men and 33 women) (Table 2).

### 3.2. Biochemical Characterization

The patients’ biochemical traits included markers for type 2 diabetes, such as free fasting glucose, HB1AC, and lipid profiles. The lipid profiles for HDL, TAGs LDL, and cholesterol were determined using colorimetric estimates (Cobas Integra 800; Roche).

### 3.3. Statistical Comparisons of T2D Patients and Controls for the HNF-1 rs1169288 G>T, miR-27a rs895819 A>G, and miR-146 rs2910164 C>G Genotypes

#### 3.3.1. Association of HNF-1α rs1169288 G>T (Ile27Leu) Genotypes with T2D

**Figure 4:**
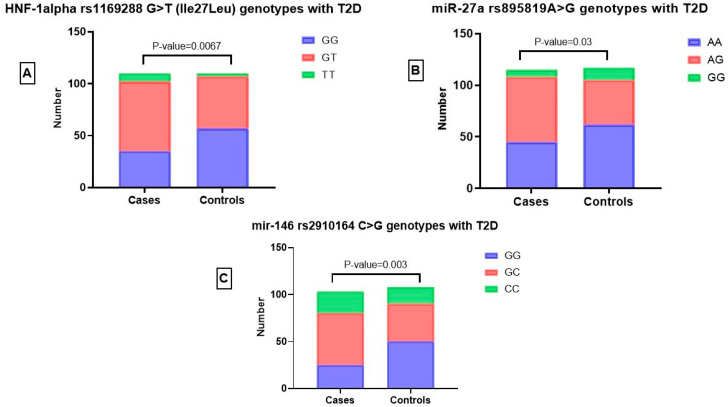
The SNVs genotype frequency in T2D patients and healthy controls. (**A**): Among the T2D cases, the genotype distribution of HNF-1 alpha rs1169288 G>T for GG, GT, and TT was 31.81, 60.90, and 7.27%, respectively. In the controls, the genotype distribution was 51.81, 45.45, and 2.72, respectively (*p*-value = 0.0067). (**B**): Among the T2D cases, the genotype distribution of miR-27a rs895819 A>G for AA, AG, and GG was 39.13, 56.52, and 6.08%, respectively. In the controls, the genotype distribution was 52.99, 36.75, and 10.25, respectively (*p*-value = 0.03). (**C**): Among the T2D cases, the genotype distribution of mir-146 rs2910164 C>G for GG, GC, and CC was 24.27, 53.39, and 22.33%, respectively. In the controls, the genotype distribution was 46.29, 37.03, and 16.66, respectively (*p*-value = 0.003).

Our results indicate significant differences in the occurrence of HNF-1α G>T (Ile27Leu) GG, TT, and GT genotypes between T2D cases and controls (*p* = 0.006) (Figure 4). Specifically, the incidence of the TT homozygote was higher in the patients (7.27%) than in the controls (2.72%), while the GG homozygote was more prevalent in the controls (51.81%) than in the patients (31.81%). We also found that the T allele was more prevalent among T2D patients (0.38%) than the controls (25%), indicating that it is a risk allele, while the G allele acted as a protective factor (Figure 4).

#### 3.3.2. Relationship between miR-27a rs895819 A>G Genotypes and T2D

According to our findings, there are statistically significant differences between T2D patients and healthy controls in the prevalence of the miR-27a A>G, AA, AG, and GG genotypes (*p* = 0.03). (Figure 4). Specifically, the incidence of the AG heterozygote was higher in T2D patients (56.52%) than in the controls (36.75%), while the AA homozygote was more frequent in the controls (52.99%) than in the T2D patients (39.13%). Similarly the frequency of the GG homozygote was higher in the controls (10.25%) than in the T2D (6.08%) patients; the G allele was more prevalent among the T2D patients (0.34) than the controls (0.28), indicating that it is a risk allele, while the A allele acted as a protective factor. (Figure 4).

Figure 4: The distribution of SNV genotypes in T2D cases and in healthy controls.

#### 3.3.3. Relationship between miR-146 rs2910164 C>G Genotypes and T2D

Our results indicate significant differences in the occurrence of miR-146 rs2910164 CC, CG, and GG genotypes between T2D cases and controls (*p* = 0.003). (Figure 4). Specifically, the incidence of the CC homozygote was higher in the patients (22.33%) compared to the controls (16.66%), while the GG homozygote was more prevalent in the controls (46.29%) compared to the patients (24.27%). We also found that the C allele was more prevalent among T2D patients (0.49) compared to the controls (0.35), indicating that it is a risk allele, while the G allele acted as a protective allele. (Figure 4).

### 3.4. Logistic Regression Analysis to Determine Association between HNF-1alpha rs1169288 G>T (Ile27Leu) Genotypes and Susceptibility to T2D

The variance in the HNF-1alpha-GT genotype was found to be significantly linked with T2D susceptibility in the codominant inheritance model, with an OR of 2.18 (95% CI) (1.2491 to 3.8127) and a RR of 1.44 (1.1135 to 1.8876) and *p*-0.0061, respectively. (Table 3). The HNF-1 alpha-TT genotype was also significantly associated with T2D susceptibility, with ORs of 4.34 (95% CI) (1.0794 to 17.4722) and 2.27 (0.8541 to 6.0423) and *p* = 0.038, respectively. The HNF-1 alpha-GG and HNF-1 alpha (GT+TT) genotypes were strongly related with T2D susceptibility in the dominant inheritance model, with odd ratios of 2.30 (95% CI) (1.3316 to 3.9885) and 1.49 (1.1526 to 1.9425) and *p* = 0.0029, respectively. The HNF-1-TT genotype was also not linked to T2D susceptibility in the recessive inheritance model, with ORs of 2.79 (95% CI) (0.7220 to 10.8379) and 1.87 (0.7087 to 4.9721) and *p* = 0.136. The allelic comparison revealed a substantial association between the HNF-1-T allele and T2D susceptibility, with ORs of 1.77, (95% CI) (1.1800 to 2.6677), RRs of 1.35, and *p*-0.005 (Table 3).

### 3.5. Association between HNF-1α rs1169288 G>T (Ile27Leu) Genotypes and the Clinicopathological Characteristics of the T2D Patients

Both male and female T2D patients had genotype distributions for HNF-1 rs1169288 G>T (Ile27Leu) that were clinically significant (*p* = 0.021) (Table 4). There was no significant relationship between the ages of the T2D patients and the HNF-1 rs1169288 G>T (Ile27Leu) genotypes (*p* = 0.071); this is in line with our previous findings (Table 4). Fasting blood glucose levels and HNF-1 rs1169288 G>T (Ile27Leu) genotypes were strongly correlated (*p* = 0.0006) (Table 4). Additionally, our results showed that patient HbA1c% and HNF-1 genotypes were strongly correlated (*p* = 0.003). There was no association between triglyceride, cholesterol, or LDL-C levels and the HNF-1 genotype. However, there was a significant variation in the HNF-1 genotype between those with low HDL-C mg/dL and people with normal HDL-C mg/dL (Table 4).

### 3.6. Multivariate and Ordinal Regression Risk Factor Analysis for T2D with miR-27a rs895819 A>G Genotypes

In the codominant model, the results suggested a potential link between the miR-27a-GA genotype and increased T2D susceptibility, with an OR = 2.01, (95% CI) (1.169 to 3.483), RR = 1.42, and *p* = 0.011 (Table 5). In the dominant inheritance model, the miR-27a-AA and (GA + GG) genotypes were strongly correlated with an increased risk of T2D, with OR = 1.70 (95% CI) (1.009 to 2.87), RR = 1.29, and *p* = 0.046 (Table 5). In the recessive inheritance, there was no discernible difference between the miR-27a—(AA+GA) and miR-27a-GG genotypes. With an OR 1.22 (95% CI) (0.822 to 1.817), RR 1.10, and *p* = 0.32, the miR-27a G allele was not associated with T2D susceptibility when allelic comparisons were made.

### 3.7. Association between miR-27a-rs895819 G>A Genotypes and the Clinicopathological Characteristics of the T2D Patients

Between male and female T2D patients, there was no discernible difference in the genotype distribution of miR-27a at rs895819 A>G (*p* = 0.101). The age of T2D patients and the MirR-27a genotypes did not significantly differ (*p* = 0.46); this is in line with our findings (Table 6). Fasting blood glucose levels and the miR-27a rs895819 A>G genotype were strongly correlated (*p* = 0.037). However, our findings indicated a significant correlation (*p* = 0.009) between HNF-1 genotypes and HbA1c%.

### 3.8. Logistic Regression Analysis of miR-146 rs2910164 C>G Genotypes to Predict Risk of T2D

The polymorphism in the miR-146 CG genotype was found to be significantly associated with T2D susceptibility in the codominant inheritance model, with ORs of 2.75 (95% CI) (1.465 to 5.161) and RRs of 1.58 (1.190 to 2.105) and *p* = 0.0061, respectively (Table 7). The miR-146-CC genotype also showed a high association with T2D susceptibility, with OR = 2.55 (95% CI) (1.169 to 5.584) and RR = 1.53 (1.037 to 2.223) and *p* = 0.038, respectively.

The miR-146-GG and miR-146 (GC+CC) genotypes were strongly related with T2D susceptibility in the dominant inheritance model, with odds of 2.68 (95% CI) (1.493 to 4.843) and 1.56 (1.214 to 2.011), respectively (Table 8). The miR-146-(GG+GC) and miR-146-CC genotypes were not linked to T2D susceptibility in the recessive inheritance model, with ORs of 1.43 (95% CI) (0.72 to 2.855) and 1.20 (0.82 to 1.75) and *p* = 0.30, respectively. The allelic comparison revealed a significant association between the miR-146-C allele and T2D susceptibility, with an OR of 1.77, (95% CI) (1.198 to 2.61), RR = 1.33, and *p* = 0.004 (Table 7).

### 3.9. Association between miR-146 rs2910164 C>G Genotypes and the Clinicopathological Characteristics of the T2D Patients

Between male and female T2D patients, there was no discernible difference in miR-146 C>G genotype distribution (*p* = 0.301). The age of the T2D patients and the miR-146 C>G genotypes were not significantly correlated (*p* = 0.57) (Table 8). Blood glucose levels during fasting were significantly correlated with MiR-146 C>G genotypes (*p* = 0.005). However, our results showed a significant association between the miR-146 C>G genotype and HbA1c% (*p* = 0.001). Triglycerides mg/dL, LDL levels, HDL levels, and cholesterol mg/dL did not correlate with one another. (Table 8).

## 4. Discussion

DM is an important issue in healthcare systems worldwide because it has very serious health and economic impacts [37]. For the current study, we evaluated the associations between the SNVs of the genes miR-27a, miR-146, and HNF-1 alpha rs1169288 G>T and T2D susceptibility among the Saudi population.

Our results indicated that the HNF-1 alpha rs1169288 G>T (Ile27Leu) T allele and TG genotype were associated with T2D (Table 3 and Table 4) and that blood sugar (HbA1c and glucose) and HDL-C levels were significantly associated with the HNF-1alpha rs1169288 G>T (Table 4). It has been reported that *HNF1* alpha gene p.I27L gene variation decreases the transcription of genes involved in the metabolism of glucose (e.g., GLUT2, HNF 4alpha, collectrin, hepatocytes pyruvate kinase, and liver growth factor activator), which induces insulin resistance and beta cell dysfunction [28]. Moreover, *HNF1* alpha knockout animals have exhibited decreased glucose transport, glucose metabolism, and mitochondrial metabolism [38].

Our results are in agreement with those reported by Morita et al., who showed that the HNF-1alpha p.I27L (rs1169288) G>T SNV is linked to T2D in the Japanese population [39]. They are also consistent with studies that have reported an association between the HNF-1 alpha rs1169288 G>T (Ile27Leu) and maturity-onset diabetes of the young (MODY) in the Turkish population and several European populations [28,38]. MODY is a monogenic disorder that occurs at around the age of 25; it is caused by defective glucose transport and metabolism as well as pancreatic beta cell dysfunction [40]. To the best of our knowledge, this is the first study to report a link between HNF-1 alpha rs1169288 G>T (Ile27Leu) SNP and T2D in the Saudi population.

Our results showed that the miR-27a-rs895819 AG genotype was linked to an elevated risk of T2D (Table 5 and Table 6). In support of this result, we also obtained significant differences in miR-27a-rs895819 genotype distribution in cases with increased fasting blood glucose and HbA1c and cases with normal fasting blood glucose and HbA1c (Table 6). This result is partially in agreement with a study by Zhu et al., who demonstrated the association between miR-27a rs895819 SNV and risk to T2D in Chinese males [41]. Elevated blood levels of miR-27a in T2D patients have been reported [42], and mir-27a expression reportedly increases in adipose tissues (cell culture) with insulin resistance in obese mice fed according to a high-fat diet [21]. Moreover, in [21], the downregulation of miR-27a in the adipocytes and in the mice promoted the uptake of sugar [21]. Furthermore, it was shown that the peroxisome proliferator-activated receptor gamma (PPAR-gamma) is a direct target of miR-27a and that the downregulation of miR-27a enhances the expression of GLUT4 and PI3K/Akt signaling via regulating PPAR-gamma expression [21].

PPAR-gamma is a transcriptional factor that is overexpressed in adipose tissues and has an important role in lipid metabolism and insulin sensitivity [43]. PI3K/AKT signaling and GLUT4 promote the uptake of sugar and are involved in the insulin signaling pathway [44]. We hypothesize that rs895819 SNV influences mir-27a expression and results in the downregulation of PPAR-gamma, which dysregulates PI3K/Akt signaling and GLUT4, resulting in insulin resistance and T2D.

Our results also show that there were significant differences (*p*-values < 0.05) in Mir-27a rs895819 genotype distribution in cases with normal and abnormal LDL-C and HDL-C blood levels (Table 6). This result is in agreement with a study that reported that Mir-27a regulates the genes of the enzymes involved in the synthesis of cholesterol, including the HMGCR gene [45]. Our results indicated that there was no significant difference in Mir-27a rs895819 genotype distribution between patients aged ≤40 and patients age ≥40. Age had no observable effect on the T2D cases carrying Mir-27a rs895819 SNV (Table 6). This result is in disagreement with a study conducted by Zhu et al., 2020 [41]. This disagreement may be because Zhu et al. examined a different age range (60 years) or because of the different populations under study.

The site of the miR-146 rs2910164 C>G SNV and its potential link to coronary artery disease were presented in a recent study [11].

Our results indicated that the CG genotype and C allele of the miR-146 rs2910164 were linked to increased susceptibility to T2D (Table 7 and Table 8). The downexpression of miR-146a is reportedly linked to the development of T2D [46]. The C allele of the rs2910164 SNV decreases mature miR-146a expression [47]. MiR-146a has been reported to directly regulate the toll/IL-1 receptors (TLRs) [48]. The TLRs, IRAK1, and TRAF6 have been shown to be involved in innate immunity and the induction of metabolic syndromes such as insulin resistance and T2D [46,49,50,51]. Furthermore, miR-146 is involved in the metabolism of sugar and lipids by regulating MED1 and directly targeting NPR3 [25,52]. The MED1 is important for lipid metabolism, and it is involved in mitochondrial function [52,53,54], whereas NPR3 regulates the uptake of sugar induced by insulin and de novo lipogenesis [25].

Therefore, our results are consistent with the above-mentioned studies, as this SNV (rs2910164 SNV) causes reduced mature miR-146 [47]. However, we did not find significant differences in rs2910164 C>G SNV between T2D cases with normal lipid profiles and T2D cases with dyslipidemia (Table 7). Perhaps this is due to the relatively small sample size of the present study.

The weaknesses of this study include its limited sample size and the fact that it is a cross-sectional one. Therefore, it is likely that the normal blood chemistry of the T2D patients was maintained after the samples were collected. Another limitation of the present study is the fact that the role of SNVs of other microRNAs in the development of insulin resistance and T2D was not investigated. The role of SNVs of other microRNA in the induction of T2D should be investigated in future studies. Examples of these microRNAs include mir-126 (reportedly decreased in T2D [55]), miR-486, miR-146b, miR-15b (involved in the development of T2D [56]), miR-197, miR-23a, miR-509-5p (contributes to metabolic syndrome [57]), miR-23a (indicated as a T2D biomarker [58]), miR-133a-1, let-7a-1 (SNVs reported to be associated with T2D [41] and others [58]). In addition, in the future, we recommended conducting longitudinal large-scale studies in different populations to further verify the diagnostic significance of our findings. Once these findings are verified in future studies, they can be used in genetic testing for stratification and comprehensive risk assessment to identify highly susceptible populations and aid in the development of precision medicine to personalize the prevention and management of T2D [59,60]. It has been reported that T2D can be prevented with strategies that involve the modification of one’s diet and behavior, regular exercise, weight control, lifestyle intervention programs, and adherence to the advice of health care professionals [61,62].

## 5. Conclusions

We conclude that the SNVs in HNF-1 alpha rs1169288 G>T, miR-27a rs895819 A>G, and miR-146 rs2910164 C>G can be potential loci for T2D risk. There was also an association between HNF-1 alpha, mirR-27a, miR-146 genotypes, HbA1c% (*p* < 0.05), and the fasting blood sugar levels of T2D patients. Moreover, there was a significant correlation between miR-27a genotypes with triglycerides (TG), LDL-C levels, and the HDL-C of the T2D patients. Future large-scale studies are needed to verify these findings. These findings can be used in genetic testing for the early identification of susceptible individuals and the prevention and treatment of insulin resistance and T2D.

## Figures and Tables

**Table 1:** Amplification Refractory Mutation System PCR primers for the genotyping of *HNF1 alpha* rs1169288 A>C (I27L), miR-27a rs895819 A>G, and miR-146ars2910164 C>G.

Amplification Refractory Mutation System PCR Primers *HNF1A* (rs1169288) A>C (I27L) SNP				
*HNF1A-Fo*		5′-GTGCCCACAGGGCTTGGCTAG-3′	387 bp	62 °C
*HNF1A-Ro*		5′-CCATCGTCGTCCGTCTCGTCCTCG-3′		
*HNF1A-FI*	(G allele)	5′-GGGCTGAGCAAAGAGGCACCG-3′	176 bp	
*HNF1A-RI*	(A allele)	5′-CCCGGCTCACCCAGTGCCTGAAT-3′	257 bp	
ARMS primers for miR27a G>A gene variation				
miR-27a-Fo		5′-GGC TTG ACC CCT GTT CCT GCT GAA CT-3′	353 bp	63.5 °C
miR-27a-Ro		5′-TTG CTT CCT GTC ACA AAT CAC ATT GCC A-3′		
miR-27a-FI	(G allele)	5′-GGA ACT TAG CCA CTG TGA ACA CGA CTT TGC-3′	184 bp	
miR-27a-RI	(A allele)	5′-CTT AGC TGC TTG TGA GCA GGG TCC CCA-3′	226 bp	
Amplification Refractory Mutation System PCR primers for miR146a-rs2910164 C>G SNP				
miR146a Fo		5′-GGC CTG GTC TCC TCC AGA TGT TTA T-3′	364 bp	61.5 °C
miR146a Ro		5′-ATA CCT TCA GAG CCT GAG ACT CTG CC-3′		
miR146a FI	(C allele)	5′-ATG GGT TGT GTC AGT GTC AGA CCT C-3′	169 bp	
miR146a RI	(G allele)	5′-GAT ATC CCA GCT GAA GAA CTG AAT TTC AC-3′	249 bp	

**Table 2:** Demographic characteristics of T2D patients.

Clinical Features	N=	%
	115	
Male	82	71.30%
Female	33	28.70%
Age > 40	91	79.13%
Age < 40	24	20.87%
FBG < 100 mg/dL	24	20.87%
FBG > 100 mg/dL	91	79.13%
HBA1c > 6%	90	78.26%
HBA1c < 6%	25	21.74%
Triglycerides mg/dL < 200	39	33.91%
Triglycerides mg/dL > 200	76	66.09%
Cholesterol mg/dL < 200	70	60.87%
Cholesterol mg/dL > 200	45	39.13%
LDL-C mg/dL < 100	57	49.57%
LDL-C mg/dL > 100	58	50.43%
HDL-L mg/dL < 55	48	41.74%
HDL-L mg/dL > 55	67	58.26%

**Table 3:** Logistic regression analysis of HNF-1alpha rs1169288 G>T (Ile27Leu) genotypes to predict T2D susceptibility.

Genotypes	Healthy Controls (N = 110)	T2D Cases (N = 110)	Odd Ratio OR (95% CI)	Risk Ratio RR (95% CI)	*p*-Value
Codominant Inheritance model					
HNF-1α-GG	57	35	Ref	Ref	
HNF-1α-GT	50	67	2.18(1.2491 to 3.8127)	1.44(1.1135 to 1.8876)	**0.0061**
HNF-1α-TT	03	08	4.34(1.0794 to 17.4722)	2.27(0.8541 to 6.0423)	**0.038**
Dominant Inheritance model					
HNF-1α-GG	57	35	Ref	Ref	
HNF-1α-(GT+TT)	53	75	2.30(1.3316 to 3.9885)	1.49(1.1526 to 1.9425)	**0.0029**
Recessive Inheritance model					
HNF-1α-(GT+GG)	107	102	Ref	Ref	
HNF-1α-TT	03	08	2.79(0.7220 to 10.8379)	1.87(0.7087 to 4.9721)	0.136
Allele					
HNF-1α-G	164	137	Ref	Ref	
HNF-1α-T	56	83	1.77(1.1800 to 2.6677)	1.35(1.0775 to 1.6974)	**0.0059**
Over dominant Inheritance model					
HNF-1α-(GG+TT)	60	43	Ref	Ref	
HNF-1α-(GT)	50	67	1.86(1.0937 to 3.1964)	1.36(1.0448 to 1.7784)	**0.022**

*p*-values lower than 0.05 are regarded as statistically significant. Bold numerals denote significant differences.

**Table 4:** Association between HNF-1alpha rs1169288 G>T genotypes and the clinicopathological characteristics of the T2D patients.

Clinical Feature	N=	GG	GA	AA	X2	DF	*p*-Value
Association of HNF-1α SNV with Gender							
Male	80	20(25%)	55(68.75%)	05(6.25%)	7.67	2	**0.021**
Female	30	15(50%)	12(40%)	3(10%)			
Association of HNF-1α SNV with Age							
>40	78	23(29.48%)	49(62.82%)	06(7.69%)	0.68	2	0.711
<40	32	12(37.5%)	18(56.25%)	02(6.25%)			
Association of HNF-1 alpha SNV with Fasting glucose mg/dL							
<100	21	13(61.90%)	5(23.80%)	03(14.28%)	15	2	**0.0006**
>100	89	22(24.71%)	62(69.66%)	5(5.61%)			
Association of HNF-1alpha SNV with HbA1c %							
>6	88	22(25%)	60(68.18%)	5(5.68%)	11.4	2	**0.003**
<6	22	13(59.09%)	7(31.81%)	3(13.63%)			
Association of HNF-1alpha SNV with Triglycerides mg/dL							
<200	77	22(28.57%)	50(64.93%)	05(6.49%)	1.75	2	0.41
>200	33	13(39.39%)	17(51.51%)	03(9.09%)			
Association of HNF-1alpha SNV with Cholesterol mg/dL							
<200	70	19(27.14%)	48(68.57%)	03(4.28%)	5.54	2	0.062
>200	40	16(40%)	19(47.5%)	05(12.5%)			
Association of HNF-1alpha SNV with LDL-C mg/dL							
<100	57	16(28.07%)	56(98.24%)	05(8.77%)	0.99	2	0.609
>100	53	19(35.84%)	31(58.49%)	03(5.66%)			
Association of HNF-1alpha SNV with HDL-L mg/dL							
<55	44	10(22.72%)	33(75%)	01(2.27%)	6.82	2	**0.033**
>55	66	25(37.87%)	34(51.51%)	07(10.60%)			

*p*-values lower than 0.05 are regarded as statistically significant. Bold numerals denote significant differences.

**Table 5:** Multivariate (logistic regression) analysis of miR-27a rs895819 A>G genotypes to evaluate susceptibility to T2D.

Genotypes	Healthy Controls (N = 117)	T2D Cases N = 115	OR (95% CI)	Risk Ratio (RR)	*p*-Value
Codominant model					
miR-27a-AA	62	45	1 Ref	1 Ref	
miR-27a-AG	43	63	2.01(1.169 to 3.483)	1.42(1.0706 to 1.899)	**0.011**
miR-27a-GG	12	07	0.80(0.2935 to 2.206)	0.91(0.6279 to 1.347)	0.67
Dominant model					
miR-27a-AA	62	45	1 Ref	1 Ref	
miR-27a-(GG+GA)	55	68	1.70(1.009 to 2.870)	1.29(1.009 to 1.674)	**0.046**
Recessive model					
miR-27a-(GA+AA)	105	108	1 Ref	1 Ref	
miR-27a-GG	12	07	0.56(0.215 to 1.493)	0.78(0.539 to 1.1294)	0.25
Allele					
miR-27a-A	167	153	1 Ref	1 Ref	
miR-27a-G	67	75	1.22(0.822 to 1.817)	1.10(0.902 to 1.355)	0.32

*p*-values lower than 0.05 are regarded as statistically significant. Bold numerals denote significant differences.

**Table 6:** Association between miR-27a genotypes and clinicopathological variables among the T2D patients.

Variable	N=	AA	AG	GG	X2	DF	*p*-Value
	115	45	63	07			
Association of miR-27a SNP with Gender							
Male	82	28(34.14%)	50(60.97%)	4(4.87%)	4.5	2	0.101
Female	33	17(51.51%)	13(39.39%)	3(9%)			
Association of miR-27a SNP with Age							
Age > 40	91	33(36.16%)	52(57.14%)	6(6.59%)	1.54	2	0.46
Age < 40	24	12(42.85%)	11(45.83%)	1(4.16%)			
Association of miR-27a SNP with FBG mg/dL							
FBG < 100 mg/dL	24	10(41.66%)	10(41.66%)	4(16.66%)	6.53	2	**0.037**
FBG > 100 mg/dL	91	35(38.46%)	53(58.24%)	3(3.29%)			
Association of miR-27a SNP with HBA1c%							
HBA1c > 6%	90	28(31.11%)	57(63.33%)	05(5.55%)	15.52	2	**0.009**
HBA1c < 6%	25	17(68%)	6(24%)	2(8%)			
Association of miR-27a SNP with Triglycerides mg/dL							
Triglycerides mg/dL < 200	39	23(58.97%)	13(33.33%)	3(7.69%)	11.14	2	**0.003**
Triglycerides mg/dL > 200	76	22(28.94%)	50(65.78%)	4(5.26%)			
Association of miR-27a SNP with Cholesterol mg/dL							
Cholesterol mg/dL < 200	70	24(34.28%)	43(61.42%)	3(4.28%)	3.47	2	0.176
Cholesterol mg/dL > 200	45	21(46.66%)	20(44.44%)	4(8.88%)			
Association of miR-27a SNP with LDL mg/dL							
LDL mg/dL < 100	57	15(26.31%)	39(68.42%)	3(5.26%)	8.71	2	**0.012**
LDL mg/dL > 100	58	30(51.72%)	24 (41.37%)	4(6.89%)			
Association of miR-27a SNP with HDL-L mg/dL							
HDL-L mg/dL < 55	48	12(25%)	33(68.75%)	3(6.25%)	7.14	2	**0.02**
HDL-L mg/dL > 55	67	33(49.25%)	30(44.77%)	4(5.97%)			

*p*-values lower than 0.05 are regarded as statistically significant. Bold numerals denote significant differences.

**Table 7:** Multivariate analysis of miR-146 rs2910164 C>G genotypes to predict the risk of T2D.

Genotypes	Healthy Controls	T2D Cases	OR (95% CI)	Risk Ratio (RR)	*p*-Value
	(N = 108)	(N = 103)			
Codominant					
miR146-GG	50	25	1(reference)	1(reference)	
miR146-GC	40	55	2.75(1.465 to 5.161)	1.58(1.190 to 2.105)	**0.0016**
miR146-CC	18	23	2.55(1.169 to 5.584)	1.53(1.037 to 2.223)	**0.0186**
Dominant					
miR-46-GG	50	25	1(reference)	1(reference)	
miR-146-(GC+CC)	58	78	2.68(1.493 to 4.843)	1.56(1.214 to 2.011)	**0.0005**
Recessive					
miR-146-(GG+GC)	90	80	1(reference)	1(reference)	
miR-146-CC	18	23	1.43(0.72 to 2.855)	1.20(0.82 to 1.75)	0.300
Allele					
miR-146-G	140	105	1(reference)	1(reference)	
miR-146-C	76	101	1.77(1.198 to 2.618)	1.33(1.088 to 1.627)	**0.004**

*p*-values lower than 0.05 are regarded as statistically significant. Bold numbers indicate significant differences.

**Table 8:** Association between miR-146 C>G Genotypes and the clinicopathological characteristics of the T2D Patients.

Clinical Feature	N=	AA	AG	GG	X2	DF	*p*-Value
Association of miR-146 SNV with Gender							
Male	70	20(28.57%)	36(51.42%)	14(20%)	2.35	2	0.301
Female	33	5(15.15%)	19(57.57%)	9(27.27%)			
Association of miR-146 SNV with Age							
>40	79	20(25.31%)	40(50.63%)	19(24.05%)	1.09	2	0.579
<40	24	5(20.83%)	15(62.5%)	4(16.66%)			
Association of miR-146 SNV with Fasting blood glucose (FBG) mg/dL							
<100	24	3(12.5%)	10(41.66%)	11(45.83%)	10.33	2	**0.005**
>100	79	22(27.84%)	45(56.96%)	12(15.18%)			
Association of miR-146 SNV with HBA1c%							
>6	78	19(24.35%)	48(61.53%)	11(14.10%)	13.73	2	**0.001**
<6	25	06(24%)	07(28%)	12(48%)			
Association of miR-146 SNV with Triglycerides mg/dL							
<200	39	12(30.76%)	15(28.46%)	12(30.76%)	5.72	2	0.057
>200	64	13(20.31%)	40(62.5%)	11(17.18%)			
Association of miR-146 SNV with Cholesterol mg/dL							
<200	58	15(25.86%)	27(46.55%)	16(27.58%)	2.95	2	0.22
>200	45	10(22.22%)	28(62.22%)	7(15.55%)			
Association of miR-146 SNV with LDL-C mg/dL							
<100	47	13(27.65%)	22(46.80%)	12(25.53%)	1.51	2	0.47
>100	56	12(21.42%)	33(58.92%)	11(19.64%)			
Association of miR-146 SNV with HDL-L mg/dL							
<55	48	11(22.91%)	27(56.25%)	10(20.83%)	0.3	2	0.86
>55	55	14(25.45%)	28(50.90%)	13(23.63%)			

*p*-values lower than 0.05 are regarded as statistically significant. Bold numerals denote significant differences.

## Data Availability

All data supporting the reported results can be found in the Prince Fahad Bin Sultan Chair for Biomedical Research, Faculty of Applied Medical Sciences, University of Tabuk, Tabuk, Saudi Arabia.

## Abbreviations

DM: diabetes mellitus; T2D: type 2 diabetes; SNV: single-nucleotide variation; ARMS–PCR: Amplification Refractory Mutation System; MiRNAs: MicroRNAs; CVD: cardiovascular diseases; HCs: healthy controls; OR: odds ratio; RR: risk ratio; CL: confidence intervals; KSA: Kingdom of Saudi Arabia; LDL-C: low-density lipoprotein cholesterol; HDL-C: high-density lipoprotein cholesterol.
